# Killing the killers

**DOI:** 10.7554/eLife.04168

**Published:** 2014-09-02

**Authors:** Marianne De Paepe, Marie-Agnès Petit

**Affiliations:** 1 **Marianne De Paepe** is in the French National Institute for Agricultural Research and AgroParisTech, Micalis Institute, Jouy-en-Josas, France; 2 **Marie-Agnès Petit** is in the French National Institute for Agricultural Research and AgroParisTech, Micalis Institute, Jouy-en-Josas, Francemarie-agnes.petit@jouy.inra.fr

**Keywords:** Vibrio cholerae, cholera, bacteriophage, phage predation, OmpU, ToxR, viruses, other

## Abstract

The bacteria that infect humans and cause cholera are themselves infected by viruses, which have the potential to influence the course of a cholera infection.

**Related research article** Seed KD, Yen M, Shapiro BJ, Hilaire IJ, Charles RC, Teng JE, Ivers LC, Boncy J, Harris JB, Camilli A. 2014. Evolutionary consequences of intra-patient phage predation on microbial populations. *eLife* **3**:e03497. doi: 10.7554/eLife.03497

**Image** Electron micrograph of a bacteriophage isolated from the stool sample of a cholera patient (adapted from Seed et al., 2011)

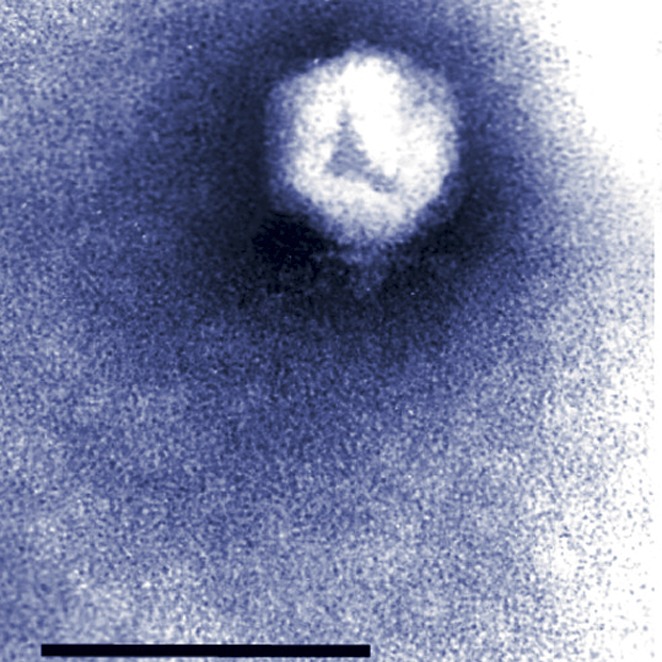

Bacteriophages are viruses that prey on bacteria. Also known as phages, they can multiply very quickly—hundreds of new viruses can be produced in a single infected bacterium in less than 30 minutes. However, relatively little is known about the impact of phage predation on human-associated bacteria in general, and even less on bacterial pathogens. Now, in *eLife*, Andrew Camilli of Tufts University School of Medicine and co-workers in Canada, Haiti and the United States, provide molecular evidence that phages prey on a bacterial pathogen during the course of an infection in humans (Seed et al., 2014).

*Vibrio cholerae* is the bacterium responsible for cholera. After being ingested, typically by drinking contaminated water, it multiplies in the digestive tract where it releases a toxin. This toxin causes profuse and watery diarrhoea, dehydration and death in 50% of cases if rehydration therapy is not administered.

In the delta region of the river Ganges in Bangladesh and India, cholera epidemics occur every year, and follow regular seasonal cycles. It has been proposed that this seasonal variation might be partly related to phages preying on the *V. cholera* bacteria—as the so-called vibriophages are most common in environmental waters at the end of the cholera season (Faruque et al., 2005a, 2005b).

Camilli and co-workers—including Kimberley Seed as the first author—report molecular data that indicate that vibriophages may preferentially prey on bacteria in the digestive tract of patients with cholera, rather than in environmental waters (Seed et al., 2014). Seed et al. looked at stool samples from two cholera patients (one from Haiti, one from Bangladesh) who had high viral loads of a type of vibriophage called ICP2. In each sample, they discovered that some of the bacteria were resistant to the phage, while the rest were sensitive to it. Then, Seed et al. sequenced the whole genomes of bacterial clones and discovered that the only differences between the phage-resistant and phage-sensitive isolates in each patient were clustered into a single gene. However, a different bacterial gene was mutated in each patient. Several different mutants of each gene were found. This strongly suggests that these mutations occurred, and were then selected for, in bacteria in the patient during the infection.

In the Haitian patient, almost all (> 99%) of the bacteria isolated were resistant to the phage; and, of the phage-resistant bacteria tested, all had one of six different mutations in a single gene called *ompU*. The OmpU protein forms a pore in the bacteria’s outer membrane to enable nutrients to be imported into the cell. The bacteria need this protein for their survival both in human hosts and in environmental waters. Since the OmpU mutants are resistant to phage attack, Seed et al.’s findings indicate that the OmpU protein is also used by the vibriophage ICP2 to infect the bacterial cells (i.e. it is also the ‘receptor’ for the ICP2 phage).

Seed et al. show that the selection of OmpU mutants by ICP2 vibriophages is not restricted to this isolated case. Out of a collection of 54 clinical isolates of *V. cholerae* collected in Bangladesh between 2001 and 2011, 15% have similar phage-resistant mutations in the *ompU* gene. Seed et al. also found that the changes in the OmpU protein were all in parts of the protein that are exposed on the outside of the bacterial cell; and importantly, that they had very little effect on the fitness of *V. cholerae* in a range of tests. This is reminiscent of the relationship between the bacterium *E. coli* and the phage lambda, where mutations in a surface protein can make the bacteria resistant to phage attack. These mutations also occur in a surface-exposed part of the protein and do not affect the other functions of this protein (Gehring et al., 1987; Hofnung, 1995). However, the *E. coli/*phage lambda studies were performed in the laboratory, whereas this *V. cholerae* study appears to be the first report that suggests a predator-prey relationship between phage and bacteria in the human intestine.

In the stool sample from the Bangladeshi patient, 22% of bacterial isolates were resistant to the ICP2 phage; and Seed et al. identified four different genetic changes that made the bacteria able to resist this phage attack. All of these mutations were in a gene called *toxR,* which encodes a protein that regulates the expression of numerous genes, including *ompU.* Since these ToxR mutants do not produce the phage’s receptor—the OmpU protein—this confers resistance to phage attack. However, the ToxR protein also regulates genes that control the virulence of the bacteria, and the ToxR mutants were unable to start new infections in an animal model of cholera. Therefore, in contrast with the OmpU mutants, it is more difficult to unambiguously assign the selection of ToxR mutants as resulting solely from defending against phage attack. Instead the selection of these non-infectious mutants could also be explained by such mutations making it ‘cheaper’ for these bacteria to grow in the digestive tract at the expense of the virulent clones.

Although it is perhaps counterintuitive, mutations that reduce virulence can have a selective advantage during an infection (Diard et al., 2013). Expressing so-called virulence proteins or factors is costly for an individual bacterium, and mutants that stop making these factors can, therefore, benefit at the expense of other bacteria that continue to do so. This advantage, however, is only short-lived as these less virulent mutants are unable to start new infections themselves. Regardless of the precise mechanism, the selection for the non-infectious ToxR mutants observed by Seed et al. suggests that phage predation may have contributed to the collapse of the infection and the selection of less virulent strains.

Finally, the results of Seed et al., together with the previous work by other groups that it builds on, highlight the important role that phages can play in shaping *V. cholerae* populations. These findings firmly place these viruses as an important ‘third’ party that must also be considered when trying to understand host–pathogen interactions.
